# Community-based sport-leisure participation and perceived community psychological resilience: a three-wave RI-CLPM study of community identification and collective efficacy

**DOI:** 10.3389/fpsyg.2026.1947539

**Published:** 2026-09-28

**Authors:** Shuai Dong, Ruoyu Li, Zexi Zhao

**Affiliations:** 1 College of Basic Course, Shanxi Institute of Energy, Jinzhong, China; 2 Department of Physical Education, Jinzhong College of Information, Jinzhong, China; 3 Department of Physical Education, Zhejiang University of Finance & Economics, Hangzhou, China

**Keywords:** collective efficacy, community identification, community-based sport-leisure participation, longitudinal mediation, perceived community psychological resilience, random intercept cross-lagged panel model (RI-CLPM)

## Abstract

**Objective:**

In modern risk societies, community psychological resilience has become an important concern for community psychology and behavioral science. Yet less is known about how everyday community-based sport-leisure participation contributes to residents’ perceived community psychological resilience over time. This study examined whether residents’ community-based sport-leisure participation predicts subsequent perceived community psychological resilience and whether this association is explained by community identification and collective efficacy.

**Methods:**

A three-wave longitudinal survey with a 4-month time lag was conducted among 386 residents in Hangzhou, China. Data were analyzed using a Random Intercept Cross-Lagged Panel Model (RI-CLPM) to separate within-person state dynamics from stable between-person differences.

**Results:**

Within-person increases in community-based sport-leisure participation positively predicted subsequent perceived community psychological resilience (T1 → T2: *β* = 0.18, *p* < 0.01; T2 → T3: *β* = 0.21, *p* < 0.01). The theory-guided standardized sequential indirect effect through community identification and collective efficacy was also significant [standardized indirect effect = 0.065, 95% CI (0.032, 0.105)].

**Conclusion:**

The findings provide longitudinal predictive evidence consistent with a behavior–identity–efficacy pathway linking residents’ sport-leisure participation with perceived community psychological resilience. Given the observational design and modest effect sizes, the pathway should be interpreted as one prospective social-psychological association rather than as evidence of a causal intervention effect. The study positions community-based sport-leisure participation as an everyday behavioral context embedded in social relationships, with implications for community-based health promotion, social connection, and resilience-related perceptions.

## 1 Introduction

In rapidly urbanizing societies, communities are increasingly understood not merely as physical residential settings but as socio-psychological ecosystems in which residents form relationships, identities, shared expectations, and collective coping resources (Kagan et al., 2024; Sen and Lei, 2024). Community psychological resilience has therefore become an important topic in community psychology and behavioral science. It refers to a community-level capacity through which residents perceive their community as able to mobilize social, emotional, organizational, and material resources when facing stress, disruption, emergencies, or structural change (Norris et al., 2008). Existing resilience research has provided important insights into disaster preparedness, governance capacity, infrastructure, and place-based vulnerability (Cutter et al., 2008; Patel et al., 2017). However, this literature has paid comparatively less attention to the everyday behavioral practices through which residents gradually construct the psychological foundations of resilient communities before crises occur.

This gap is important because community resilience is not formed only during disasters or public emergencies. Rather, it may be cultivated through ordinary interactions, repeated contact, and shared routines embedded in daily community life. Social capital research suggests that community networks, trust, reciprocity, and informal support are central to the capacity of communities to adapt and recover from adversity (Aldrich and Meyer, 2015). From this perspective, the question is not only what resources resilient communities possess, but also how residents’ routine behaviors create the social-psychological conditions under which such resources become visible and meaningful. Community-based sport-leisure participation represents a particularly relevant but underexamined behavioral context for this process.

Community-based sport-leisure participation refers to residents’ involvement in regular sport, exercise, and recreational physical activities organized or informally practiced within community spaces. Unlike individual physical activity performed in isolation, community-based sport-leisure activities often involve repeated co-presence, shared public space, mutual observation, informal coordination, and emotionally positive interaction. A growing body of research suggests that sport participation may generate psychological and social benefits beyond physical health, including social interaction, social capital, and community development (Eime et al., 2013; Misener and Mason, 2006; Nicholson and Hoye, 2008; Zhou and Kaplanidou, 2018). Moreover, social infrastructure research emphasizes that local spaces and activities that bring residents together can foster social cohesion, belonging, and subjective wellbeing (Latham and Layton, 2022; Zahnow, 2024). Therefore, community-based sport-leisure participation may function as an informal social infrastructure: it provides low-threshold, recurring opportunities for residents to encounter one another, build familiarity, and transform physical proximity into social connection. Recent Chinese research similarly links physical activity with psychological resilience and mental-health-related outcomes, including anxiety and negative emotions (Yuan et al., 2025; Hu et al., 2026; Yue et al., 2026). These studies largely focus on individual-level psychological functioning, leaving open whether participation embedded in neighborhood settings is associated with community-level resilience perceptions.

To explain how such behavioral participation may be translated into perceived community psychological resilience, the present study integrates Social Identity Theory (SIT) and Social Cognitive Theory (SCT) (Davis et al., 2019; Scott et al., 2024). Broaden-and-Build Theory is used as a complementary account for the direct H1 association, describing how positive affect arising from leisure participation may broaden social engagement and resource-related appraisals; SIT and SCT remain the core theories for the proposed sequential identity–efficacy mechanism. According to SIT, individuals’ self-concepts are partly derived from social group membership, and group identity becomes stronger when membership is made salient through shared experience, interaction, and emotional meaning (Greenwood, 1994; Hogg et al., 1995; Leach et al., 2008). In community settings, residents may share the same geographic space without necessarily developing a strong psychological connection to that community. Community-based sport-leisure participation may bridge this gap by transforming co-residence into co-presence and co-presence into shared belonging. Repeated participation in familiar sport-leisure spaces can make community membership more visible, emotionally meaningful, and psychologically internalized.

However, community identification alone may not fully explain why residents come to perceive their community as resilient. Social Cognitive Theory emphasizes agency beliefs, including collective efficacy, which refers to a group’s shared belief in its conjoint capacity to organize and execute actions required to achieve desired outcomes (Bandura, 1990, 1997, 2000). In community contexts, collective efficacy is closely linked to social cohesion, mutual trust, and expectations that neighbors will intervene or cooperate for the common good (Sampson et al., 1997). Sport-leisure participation may provide residents with small-scale but repeated experiences of rule-following, coordination, mutual help, and shared use of public resources. Through these experiences, residents may observe that neighbors are trustworthy and capable of acting together. This may strengthen collective efficacy, which in turn is theoretically relevant to perceived community psychological resilience because resilience depends not only on emotional attachment but also on confidence in collective problem-solving. Related exercise research also indicates that self-efficacy is associated with physical-activity engagement and can function as a motivational pathway linking personal resources to exercise behavior (Hu et al., 2025; Chen et al., 2026). Although individual self-efficacy is conceptually distinct from collective efficacy, these findings are consistent with the broader SCT proposition that efficacy beliefs are behaviorally consequential; the present study extends that agency perspective to shared community capability.

The present study therefore proposes a behavior–identity–efficacy pathway. In this pathway, community-based sport-leisure participation is conceptualized as a behavioral starting point; community identification reflects an affective and identity-based transformation; collective efficacy represents a cognitive and agency-based transformation; and perceived community psychological resilience is the psychological outcome of this process. This framework extends previous work by explaining how an everyday behavioral practice may be linked to community-level psychological resources through sequential social-psychological mechanisms.

Although this theoretical pathway is plausible, existing empirical evidence remains limited. Much prior research on sport participation, community identification, collective efficacy, and resilience has relied on cross-sectional designs (Morgan et al., 2019; Tang et al., 2025; Zhang et al., 2024). Cross-sectional data cannot determine whether changes in sport-leisure participation precede changes in identification, efficacy, or resilience, and they may conflate stable between-person differences with within-person changes over time. For example, residents who are generally more sociable may report higher participation, stronger community identification, and greater perceived resilience, even if changes in participation do not predict later psychological changes.

To address this limitation, this study used a three-wave longitudinal design and a Random Intercept Cross-Lagged Panel Model (RI-CLPM). The RI-CLPM separates stable between-person differences from within-person fluctuations, allowing a more stringent test of whether deviations from a resident’s own typical level of sport-leisure participation predict subsequent changes in community identification, collective efficacy, and perceived community psychological resilience (Hamaker et al., 2015; Orth et al., 2021). Thus, the central question of this study is not merely whether residents who participate more in sport-leisure activities are generally more resilient, but whether increases in a resident’s participation above their own usual level are associated with later increases in community-related psychological resources. The conceptual model is shown in Figure 1.

**Figure 1:**
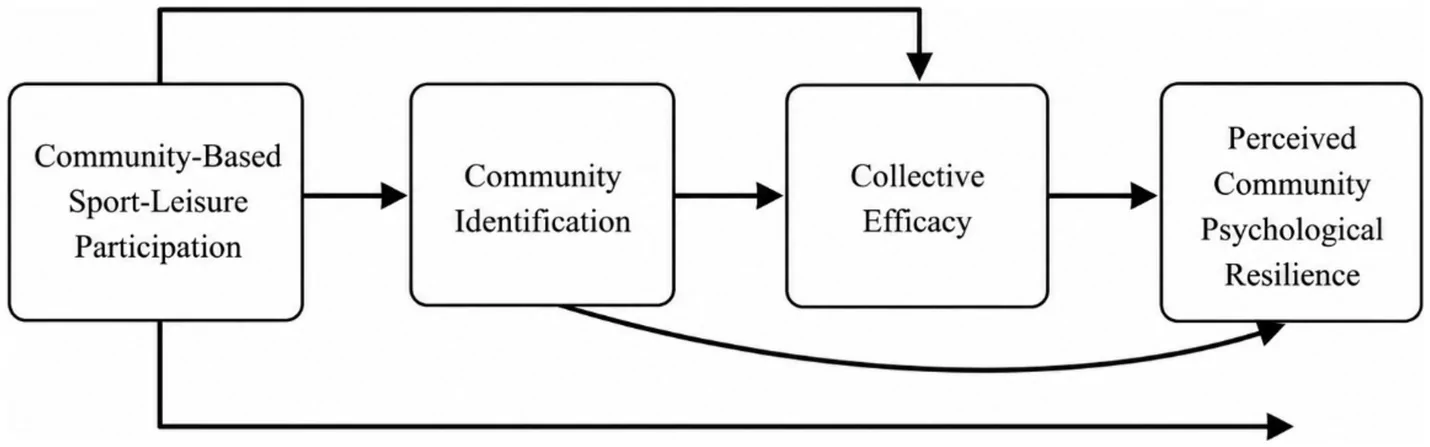
Conceptual model of the study.

## 2 Theoretical hypotheses

### 2.1 The longitudinal main effect of community-based sport-leisure participation on perceived community psychological resilience

Perceived community psychological resilience is not a fixed attribute of a place; it is shaped by residents’ appraisal of whether their community can maintain social support, mobilize resources, coordinate responses, and recover from adversity (Norris et al., 2008; Patel et al., 2017). While resilience has often been examined in relation to disaster preparedness and institutional capacity, it also depends on residents’ everyday experiences of social connection and mutual support. In urban communities, sport-leisure activities embedded in public or semi-public community spaces provide regular opportunities for residents to encounter neighbors, share routines, and experience positive emotions in familiar local settings.

According to the Broaden-and-Build Theory, positive emotions broaden individuals’ thought-action repertoires and help build enduring personal and social resources (Fredrickson, 2001). Community-based sport-leisure participation may therefore contribute to resilience perceptions through both individual and social pathways. At the individual level, participation may generate positive affect and stress relief. At the community level, it increases residents’ exposure to neighborhood interactions, shared public spaces, and informal support networks. These repeated experiences may help residents perceive their community as socially connected, emotionally supportive, and capable of coping with difficulties.

From a within-person longitudinal perspective, an increase in a resident’s sport-leisure participation above their usual level may indicate greater involvement in local interaction spaces and stronger exposure to neighborhood resources. Such deviations from one’s typical participation level may subsequently shape how residents evaluate the community’s preparedness, connectedness, and adaptive capacity. Therefore, this study expects that within-person increases in community-based sport-leisure participation will predict subsequent increases in perceived community psychological resilience.

Based on this, the present study proposes:

*H1*: A within-person increase in residents’ community-based sport-leisure participation positively predicts subsequently perceived community psychological resilience.

### 2.2 The longitudinal mediating role of community identification

Social Identity Theory posits that individuals define themselves partly through the groups to which they belong, and that such identification is strengthened when group membership becomes salient, meaningful, and emotionally valued (Greenwood, 1994; Hogg et al., 1995; Leach et al., 2008). In urban communities, residents may live in spatial proximity without developing strong community identification. Residential proximity alone does not automatically produce psychological belonging. Instead, belonging is often formed through repeated interaction, shared experience, mutual recognition, and emotional connection.

Community-based sport-leisure participation may facilitate this process by transforming passive co-residence into active co-presence. Through walking groups, running clubs, square dancing, ball games, fitness activities, or other informal sport-leisure practices, residents repeatedly encounter one another in recognizable community spaces. Such activities provide shared routines, visible participation, and opportunities for informal communication. These experiences may increase the salience of community membership and help residents reinterpret their community from a mere place of residence into a meaningful social group. This reasoning is also consistent with the sense-of-community literature, which emphasizes membership, influence, fulfillment of needs, and shared emotional connection as foundations of community belonging (McMillan and Chavis, 1986; Peterson et al., 2008).

Community identification may then support perceived community psychological resilience. Residents who feel bonded with their community are more likely to perceive social support from other members, interpret community problems as shared concerns, and believe that the community has the relational resources needed to respond to difficulties. In temporal terms, prior sport-leisure participation provides the behavioral context for the later strengthening of identification, and this strengthened identification may contribute to subsequent evaluations of community resilience.

Based on this, the present study proposes:

*H2*: Community identification mediates the longitudinal association between community-based sport-leisure participation and perceived community psychological resilience.

### 2.3 The longitudinal mediating role of collective efficacy

Collective efficacy refers to a group’s shared belief in its conjoint capability to organize and execute actions required to achieve desired outcomes (Bandura, 1990, 1997, 2000). In neighborhood research, collective efficacy is commonly understood as the combination of social cohesion, mutual trust, and shared expectations that residents will act together for the common good (Sampson et al., 1997). This construct is particularly relevant to community resilience because resilience requires not only emotional attachment to the community but also confidence in the community’s ability to coordinate and respond collectively.

Community-based sport-leisure activities may strengthen collective efficacy through repeated small-scale experiences of cooperation. Many sport-leisure practices require residents to share public spaces, follow informal rules, coordinate time and resources, negotiate minor conflicts, and observe others’ willingness to cooperate. These experiences may provide residents with concrete evidence that neighbors can act responsibly and collaboratively. From the perspective of Social Cognitive Theory, such mastery-like and observational experiences are important sources of efficacy beliefs. Although community sport-leisure activities are not formal emergency-response practices, they may function as everyday rehearsals of coordination, mutual adjustment, and collective agency.

Over time, residents who participate more actively in such activities may become more confident that neighbors will cooperate when collective action is needed. This belief is directly relevant to perceived community psychological resilience because communities are perceived as resilient when residents believe that members can support one another, organize responses, and solve problems together. Therefore, collective efficacy is expected to serve as a cognitive mechanism linking sport-leisure participation to later perceived community resilience.

Based on this, the present study proposes:

*H3*: Collective efficacy mediates the longitudinal association between community-based sport-leisure participation and perceived community psychological resilience.

### 2.4 The longitudinal sequential mediating role of community identification and collective efficacy

After examining community identification and collective efficacy as separate mediating mechanisms, this study further integrates Social Identity Theory and Social Cognitive Theory to propose a longitudinal behavior–identity–efficacy pathway. This pathway assumes that the psychological influence of sport-leisure participation unfolds through a sequence from behavioral participation to affective belonging, from affective belonging to collective agency beliefs, and finally from collective agency beliefs to perceived community psychological resilience.

From a psychological process perspective, community identification may provide the affective foundation for collective efficacy. Identification answers the question “Do I belong to this community?,” whereas collective efficacy answers the question “Can we act together as a community?” A resident may feel attached to a community without necessarily believing that community members can coordinate action. However, stronger community identification can reduce interpersonal distance, increase trust, and encourage residents to interpret neighborhood interactions as evidence of shared capability. In this way, identification may transform social familiarity into collective agency.

Community-based sport-leisure participation is expected to initiate this sequence because it creates repeated opportunities for residents to interact in meaningful local spaces. Through these interactions, residents may gradually strengthen their sense of “we-ness,” which then supports stronger beliefs that community members can work together to address shared problems. Such collective efficacy, in turn, may directly shape residents’ perceptions of whether the community is psychologically resilient. Therefore, community-based sport-leisure participation is not expected to contribute to perceived community psychological resilience only through a direct pathway; rather, its influence may operate through a gradual transformation from behavior to identity and from identity to collective efficacy.

Based on a three-wave longitudinal perspective, this study proposes the core sequential mediation hypothesis:

*H4*: Community identification and collective efficacy form a longitudinal sequential mediation pathway between community-based sport-leisure participation and perceived community psychological resilience.

## 3 Materials and methods

### 3.1 Participants and procedure

This study employed a three-wave longitudinal design, with data collected from three urban communities in Hangzhou, Zhejiang Province that had established sport-leisure facilities and regular community-based recreational sport activities. These communities were selected because they provided a suitable setting for examining residents’ everyday sport-leisure participation in relation to social-psychological community processes. To reduce common method variance and establish temporal separation, a 4-month time lag was applied between consecutive waves: baseline (T1) in March 2025, intermediate (T2) in July 2025, and outcome (T3) in November 2025. A 4-month interval was selected because it was considered long enough for changes in community interaction, identity-related perceptions, and collective efficacy beliefs to emerge, while short enough to reduce excessive attrition in a community-based longitudinal survey. Prior to study initiation, ethical approval was obtained from the Institutional Review Board (IRB) of the affiliated university, and informed consent was secured from all participants. At T1, a purposive sampling approach was used to distribute 800 questionnaires via community property management groups and offline events. To match longitudinal responses while maintaining anonymity, a self-generated identification code was adopted. After excluding responses with systematic missingness, unengaged responses, and dropouts, the final valid sample with fully matched data across T1, T2, and T3 comprised 386 participants (retention rate = 48.25%). Little’s MCAR test indicated that missing data were missing completely at random (*χ*^2^ = 34.12, df = 28, *p* > 0.05), reducing concerns about systematic attrition bias. Among the final 386 participants, 48.2% were male and 51.8% were female. Age ranged from 24 to 55 years (*M* = 35.2, SD = 8.1). Regarding educational attainment, individuals with a bachelor’s degree or above accounted for the largest proportion (50%), followed by associate degrees (30%) and high school or below (20%) (Table 1).

**Table 1:** Demographic characteristics of participants (*N* = 386).

Variables	Categories	*n*	%
Gender	Male	186	48.2
	Female	200	51.8
Age	≤30 years	116	30.1
	31–40 years	154	39.9
	41–50 years	77	19.9
	≥ 51 years	39	10.1
Education	High school or below	77	19.9
	Associate degree	116	30.1
	Bachelor’s degree	154	39.9
	Master’s degree or above	39	10.1

### 3.2 Measures

All measurement tools employed in this study were adapted from established scales published in authoritative international psychology and leisure journals. To support cross-cultural adaptation, the original English scales underwent translation and back-translation following Brislin (1970). Adaptation was deliberately conservative: wording changes were restricted to placing the original constructs in the context of residents’ urban Chinese community sport-leisure experiences, while the source constructs and 5-point response format were retained except for the explicitly noted item selection for perceived community psychological resilience. The specific contextual modifications are described for each measure below. All items were rated on a 5-point Likert scale (1 = strongly disagree, 5 = strongly agree). Reliability and validity were evaluated using Cronbach’s *α*, McDonald’s *ω*, Composite Reliability (CR), Average Variance Extracted (AVE), and the longitudinal measurement-invariance tests reported below.

#### 3.2.1 Community-based sport-leisure participation

This construct was measured using the Behavioral Involvement Scale developed by Kyle et al. (2004), adapted to the community sport-leisure context. The original behavioral involvement items were reworded to refer specifically to residents’ participation in sport, exercise, and recreational physical activities within or around their residential community. Four core items assessed the frequency and depth of participation in community-based sport-leisure activities. Example content included how often residents participated in community sport-leisure activities and the extent to which such activities were integrated into their everyday community life. Across the three waves, reliability and validity indicators were acceptable to excellent (*α* = 0.86–0.89, *ω* = 0.86–0.90, CR > 0.85, AVE > 0.58).

#### 3.2.2 Community identification

Adapted from the Multicomponent Model of In-Group Identification developed by Leach et al. (2008), utilizing 5 items from the “solidarity” and “centrality” dimensions (e.g., “I feel a bond with my community”). The indicators across the three waves were: α ranged from 0.89 to 0.91, ω ranged from 0.89 to 0.92, CR > 0.88, AVE > 0.62. In the localized wording, the respondent’s residential community was used as the group referent while the solidarity and centrality content of the selected items was retained.

#### 3.2.3 Collective efficacy

Measured using a 5-item scale adapted from Sampson et al. (1997), focusing on informal social control and social trust (e.g., “If a sudden problem arises in the community, I believe neighbors will unite to solve it”). The indicators across the three waves were: α ranged from 0.87 to 0.90, ω ranged from 0.88 to 0.91, CR > 0.88, AVE > 0.60. The adapted wording used neighbors and the residential community as the immediate referents while preserving the original emphasis on social trust, informal coordination, and shared capacity for collective action.

#### 3.2.4 Perceived community psychological resilience

This construct was measured using the Conjoint Community Resiliency Assessment Measure (CCRAM) by Leykin et al. (2013). Because the present study used residents’ self-reports, the outcome is labelled as perceived community psychological resilience. To reduce conceptual overlap with collective efficacy, items from the CCRAM collective-efficacy dimension were excluded, and six items assessing leadership, preparedness, and place attachment were retained. Across the three waves, reliability and validity indicators were excellent (*α* = 0.90–0.92, *ω* = 0.91–0.93, CR > 0.91, AVE > 0.65).

#### 3.2.5 Control variables

Gender, age, and educational level were statistically controlled in subsequent model analyses to rule out potential confounding effects on the dependent variable.

### 3.3 Data analysis strategy

Data processing and analysis were conducted using the lavaan package in R (Version 4.3.1). The analysis proceeded in three steps. First, confirmatory factor analysis (CFA) was performed to examine the discriminant validity of the four core constructs, and an Unmeasured Latent Method Factor (ULMF) approach was used to assess potential common method variance (CMV). Second, longitudinal measurement invariance was tested by sequentially evaluating configural, metric, and scalar invariance models. In accordance with Cheung and Rensvold (2002), changes in model fit satisfying ΔCFI < 0.01 and ΔRMSEA < 0.015 were considered evidence of temporal measurement stability. Third, RI-CLPM was estimated to distinguish stable between-person differences from within-person fluctuations over time (Hamaker et al., 2015). Random intercepts for the four latent variables were specified with factor loadings constrained to 1 across all time points. The residual within-person components were then used to estimate autoregressive and cross-lagged paths. Model parameters were estimated using maximum likelihood based on the fully matched longitudinal sample.

## 4 Results

### 4.1 Attrition and non-response bias analysis

In this 8-month, three-wave longitudinal study, sample attrition was expected. At T1, 800 valid questionnaires were collected, and 386 participants were retained with fully matched data across all three waves (retention rate = 48.25%). To assess potential attrition bias, we compared the retained sample (*N* = 386) with participants who dropped out at T2 or T3 (*N* = 414) based on their baseline data. Independent-samples t-tests and chi-square tests indicated no significant differences between the two groups in gender (*χ*^2^ = 1.12, *p* = 0.290), age (*t* = 0.85, *p* = 0.396), or educational level (*χ*^2^ = 2.04, *p* = 0.564). There were also no statistically significant differences in baseline scores of the core variables: community-based sport-leisure participation (*t* = 1.05, *p* = 0.295), community identification (*t* = −0.78, *p* = 0.436), collective efficacy (*t* = 1.12, *p* = 0.264), and perceived community psychological resilience (*t* = 0.94, *p* = 0.348). Combined with Little’s MCAR test (*p* > 0.05), these findings suggest that attrition was unlikely to introduce substantial systematic bias into the longitudinal analyses.

### 4.2 Descriptive statistics and correlation analysis

The means, standard deviations, reliability, and convergent validity indicators of the core variables across the three waves are shown in Table 2. The mean trajectories shown in Figure 2 indicate gradual increases in community-based sport-leisure participation, community identification, collective efficacy, and perceived community psychological resilience over the 8-month period. All constructs demonstrated acceptable to excellent psychometric properties. Cronbach’s α coefficients and Composite Reliability (CR) values ranged from 0.86 to 0.93 across time points, exceeding the recommended threshold of 0.70. Average Variance Extracted (AVE) values ranged from 0.61 to 0.68, exceeding the recommended threshold of 0.50 and supporting convergent validity.

**Table 2:** Descriptive statistics, reliability, and validity (*N* = 386).

Time/variables	*M*	SD	*α*	CR	AVE
Wave 1 (T1)					
Sport-Leisure participation	3.35	0.82	0.86	0.87	0.62
Community identification	3.42	0.78	0.89	0.89	0.64
Collective efficacy	3.38	0.75	0.87	0.88	0.61
Perceived comm. resilience	3.45	0.79	0.90	0.91	0.65
Wave 2 (T2)					
Sport-leisure participation	3.48	0.80	0.88	0.89	0.63
Community identification	3.56	0.76	0.90	0.90	0.65
Collective efficacy	3.52	0.73	0.89	0.90	0.62
Perceived comm. resilience	3.60	0.77	0.91	0.92	0.66
Wave 3 (T3)					
Sport-leisure participation	3.62	0.81	0.89	0.90	0.64
Community identification	3.70	0.75	0.91	0.92	0.67
Collective efficacy	3.68	0.71	0.90	0.91	0.65
Perceived comm. resilience	3.75	0.74	0.92	0.93	0.68

M, Mean; SD, Standard Deviation; α, Cronbach’s Alpha; CR, Composite Reliability; AVE, Average Variance Extracted.

**Figure 2:**
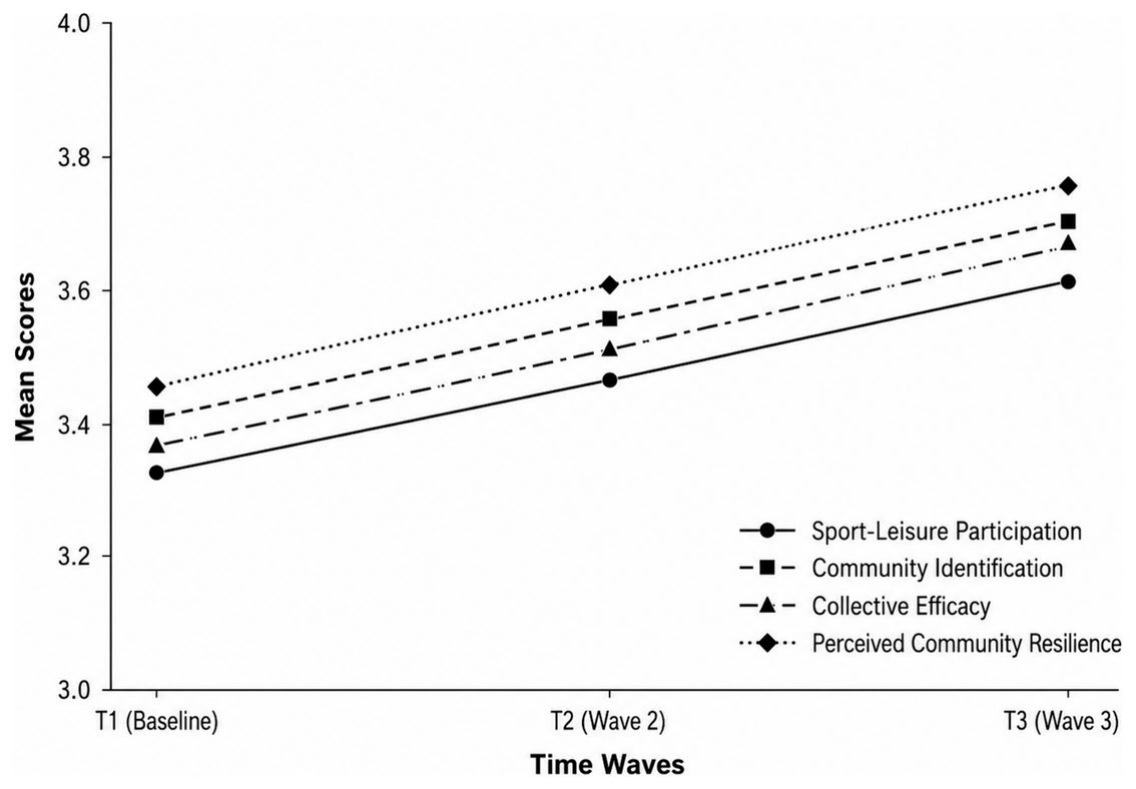
Mean trajectories of core variables across three waves.

Table 3 presents the Pearson correlation matrix among all variables. Autoregressive correlations of the same constructs at adjacent time points ranged from 0.51 to 0.66, indicating moderate temporal stability. As expected, cross-time positive correlations were observed between antecedent and subsequent variables, such as T1 sport-leisure participation and T2 community identification (*r* = 0.48, *p* < 0.01), and T2 community identification and T3 collective efficacy (*r* = 0.52, *p* < 0.01). These associations provide preliminary support for the proposed longitudinal mediation model. None of the correlation coefficients exceeded 0.75, suggesting that multicollinearity was unlikely to be a serious concern.

**Table 3:** Pearson correlation matrix across three waves (*N* = 386).

Variables	1	2	3	4	5	6	7	8	9	10	11	12
T1 SLP	1											
T1 CI	0.42	1										
T1 CE	0.38	0.45	1									
T1 PCPR	0.35	0.41	0.48	1								
T2 SLP	0.65	0.39	0.36	0.33	1							
T2 CI	0.48	0.62	0.42	0.38	0.45	1						
T2 CE	0.36	0.49	0.60	0.44	0.41	0.48	1					
T2 PCPR	0.32	0.43	0.51	0.64	0.38	0.45	0.52	1				
T3 SLP	0.52	0.34	0.31	0.29	0.66	0.42	0.38	0.35	1			
T3 CI	0.41	0.55	0.37	0.34	0.50	0.63	0.44	0.40	0.47	1		
T3 CE	0.33	0.42	0.53	0.39	0.43	0.52	0.61	0.47	0.44	0.50	1	
T3 PCPR	0.29	0.38	0.44	0.56	0.39	0.48	0.55	0.65	0.41	0.46	0.54	1

SLP, community-based sport-leisure participation; CI, community identification; CE, collective efficacy; PCPR, perceived community psychological resilience. All correlations are significant at *p* < 0.01 (two-tailed).

### 4.3 Common method bias and longitudinal measurement invariance

Considering that self-report longitudinal surveys may still be affected by common method variance (CMV), statistical checks were implemented after data aggregation. Harman’s single-factor test revealed that the first unrotated principal component explained only 32.4% of the total variance, below the empirical 40% threshold. The Unmeasured Latent Method Factor (ULMF) test further indicated that introducing a global method factor into the four-factor baseline model did not yield a substantive improvement in overall model fit (ΔCFI = 0.003, ΔRMSEA = 0.002). This suggests that CMV was unlikely to pose a substantial threat. Before applying structural equation modeling to evaluate cross-lagged effects, longitudinal measurement invariance was examined. As shown in Table 4, nested model comparisons were conducted in the sequence of configural, metric, and scalar invariance. After constraining factor loadings to be equal across time, the change in model fit indices was small (ΔCFI = −0.002, ΔRMSEA = 0.001); after further constraining intercepts, the magnitude of change remained within the criteria recommended by Cheung and Rensvold (2002) (ΔCFI < 0.01, ΔRMSEA < 0.015). The establishment of scalar invariance supports comparisons of latent variable means and structural paths across waves.

**Table 4:** Results of longitudinal measurement invariance testing.

Model	*χ* ^2^	df	CFI	TLI	RMSEA	Model Comparison	ΔCFI	ΔRMSEA
M1: configural invariance	645.23	412	0.965	0.958	0.041	–	–	–
M2: metric invariance	668.45	438	0.963	0.957	0.042	M2 vs. M1	−0.002	0.001
M3: scalar invariance	701.12	464	0.959	0.955	0.044	M3 vs. M2	−0.004	0.002

### 4.4 Alternative model comparisons

In longitudinal research, support for hypothesized lagged paths should be complemented by alternative model comparisons. We therefore compared three competing RI-CLPM models (Table 5). The reverse direction model (M2), which specified paths from perceived community psychological resilience and collective efficacy to later sport-leisure participation, showed poorer fit than the hypothesized model (CFI = 0.945, RMSEA = 0.058). This pattern was less consistent with a reverse-direction explanation. We also compared the hypothesized model (M1) with the fully reciprocal model (M3). Although M3 allowed all constructs to predict each other and showed slightly better absolute fit, it estimated additional parameters and reduced the degrees of freedom by 8. Because this increase in model complexity did not significantly improve model fit relative to M1 (Δ*χ*^2^(8) = 7.14, *p* = 0.522), the more parsimonious hypothesized model was retained (*χ*^2^ = 765.34, df = 418, CFI = 0.968, RMSEA = 0.046).

**Table 5:** Alternative model comparisons.

Models	*χ* ^2^	df	CFI	TLI	RMSEA	SRMR	Comparison	Δ*χ*^2^	Δdf	*p*-value
M1: hypothesized model (forward paths)	765.34	418	0.968	0.962	0.046	0.039	M1 vs. M3	7.14	8	0.522 (ns)
M2: reverse direction model (reverse paths)	892.15	424	0.945	0.938	0.058	0.061	–	–	–	–
M3: fully reciprocal model (bidirectional)	758.20	410	0.969	0.961	0.045	0.038	–	–	–	–

*N* = 386. M1 is the final retained model; ns, not significant (*p* > 0.05).

### 4.5 Hypothesis testing: within-person RI-CLPM results

Unlike traditional cross-lagged panel models, the RI-CLPM used in this study separates stable, trait-like between-person variance from within-person fluctuations over time. The simplified focal within-person paths of the final model are presented in Figure 3 and Table 6. For visual clarity, Figure 3 displays only the paths directly relevant to the hypotheses; the complete RI-CLPM specification, including random intercepts, autoregressive paths, cross-lagged paths, and residual covariances, is described in the Methods. Autoregressive paths were significant for all constructs, indicating that each variable showed temporal stability across the 4-month intervals even after stable between-person differences were separated.

**Figure 3:**
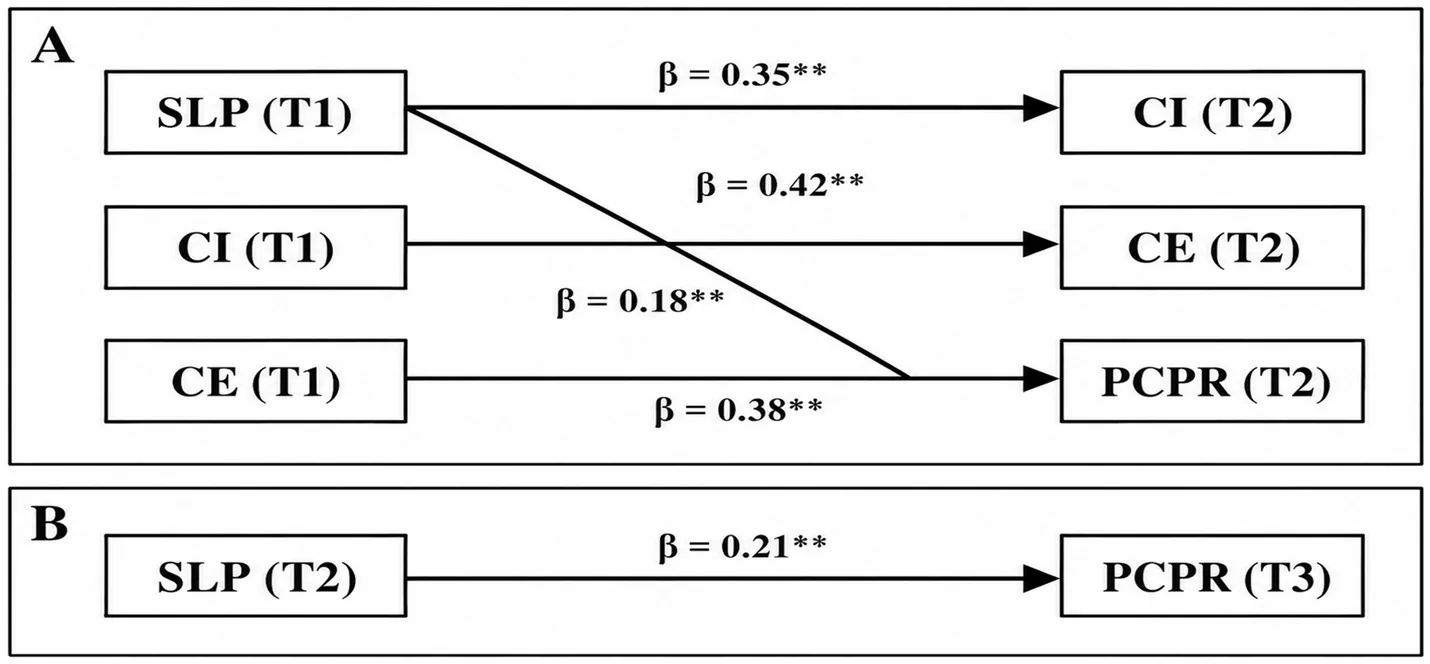
Simplified focal within-person paths in the RI-CLPM. SLP, community-based sport-leisure participation; CI, community identification; CE, collective efficacy; PCPR, perceived community psychological resilience. For visual clarity, Figure 3 displays only the focal hypothesis-relevant within-person paths, including the comparable T2-to-T3 direct path from SLP to PCPR. Random intercepts, autoregressive paths, and residual covariances are omitted from the figure for clarity and are specified in the Methods. **p < 0.01. Panel **A** shows the focal T1-to-T2 within-person paths, whereas Panel **B** shows the comparable T2-to-T3 direct path from SLP to PCPR.

**Table 6:** Standardized within-person direct paths and bootstrap indirect effects of the RI-CLPM.

Paths	Effect type	Std. estimate	SE	95% CI	Conclusion
Direct effects					
SLP (T1) → PCPR (T2)	Main	0.18**	0.04	[0.10, 0.26]	H1 supported
SLP (T1) → CI (T2)	Antecedent	0.35**	0.05	[0.25, 0.45]	—
CI (T1) → CE (T2)	Intermediate	0.42**	0.05	[0.32, 0.52]	—
CE (T1) → PCPR (T2)	Consequent	0.38**	0.04	[0.30, 0.46]	—
SLP (T2) → PCPR (T3)	Comparable interval	0.21**	—	—	Consistent with H1
Bootstrap indirect effects					
SLP (T1) → CI (T2) → PCPR (T3)	Indep. mediation	0.098*	0.025	[0.052, 0.148]	H2 supported
SLP (T1) → CE (T2) → PCPR (T3)	Indep. mediation	0.085*	0.022	[0.045, 0.130]	H3 supported
SLP (T1) → CI (T2) → CE (T2) → PCPR (T3)	Sequential med.	0.065*	0.018	[0.032, 0.105]	H4 supported

SLP, community-based sport-leisure participation; CI, community identification; CE, collective efficacy; PCPR, perceived community psychological resilience. SE, standard error; CI, confidence interval. Table 6 displays the focal standardized direct paths and standardized model-defined bootstrap indirect effects from the retained RI-CLPM. Values in the Std. Estimate column are standardized estimates throughout: direct effects are reported as standardized *β* coefficients, and indirect effects are reported as standardized bootstrap indirect effects. The additional SLP (T2) → PCPR (T3) row is included to show the comparable direct effect across the second interval displayed in Figure 3. Dashes indicate that separate SE and CI values were not additionally tabulated for that replicated interval path in this summary table. Bootstrap confidence intervals that do not include 0 indicate significant indirect effects. *p < 0.05, **p < 0.01.

#### 4.5.1 Main effect testing (H1)

After controlling for prior autoregressive effects and contemporaneous correlations, T1 community-based sport-leisure participation positively predicted T2 perceived community psychological resilience (*β* = 0.18, *p* < 0.01). A comparable positive path was also observed from T2 to T3 (*β* = 0.21, *p* < 0.01; see Figure 3 and Table 6). These results suggest that when a resident’s sport-leisure participation is higher than their own usual level, their subsequent evaluation of community psychological resilience tends to increase. Thus, H1 was supported.

#### 4.5.2 Mediation and sequential mediation testing (H2, H3, H4)

To test the longitudinal mediation mechanisms, we used a bias-corrected nonparametric percentile bootstrap procedure based on 5,000 resamples. Indirect effects were estimated at the within-person level after stable between-person differences had been separated. Figure 3 displays only the focal hypothesis-relevant paths in the main text. Table 6 reports the focal standardized direct paths and the standardized model-defined bootstrap indirect effects used for hypothesis testing.

As shown in Table 6, the focal direct path from SLP (T1) to PCPR (T2) was positive and significant [standardized *β* = 0.18, 95% CI = (0.10, 0.26)], supporting H1. A comparable direct path from SLP (T2) to PCPR (T3) was also positive and significant (standardized *β* = 0.21, *p* < 0.01). The verified direct paths reported for the retained model were likewise positive and significant: SLP (T1) positively predicted CI (T2) [standardized *β* = 0.35, 95% CI = (0.25, 0.45)], CI (T1) positively predicted CE (T2) [standardized *β* = 0.42, 95% CI = (0.32, 0.52)], and CE (T1) positively predicted PCPR (T2) [standardized *β* = 0.38, 95% CI = (0.30, 0.46)].

The standardized bootstrap indirect effect through community identification was significant [SLP (T1) → CI (T2) → PCPR (T3): standardized indirect effect = 0.098, SE = 0.025, 95% CI = (0.052, 0.148)], supporting H2. The standardized bootstrap indirect effect through collective efficacy was also significant [SLP (T1) → CE (T2) → PCPR (T3): standardized indirect effect = 0.085, SE = 0.022, 95% CI = (0.045, 0.130)], supporting H3. Finally, the standardized sequential indirect effect through community identification and collective efficacy was significant [SLP (T1) → CI (T2) → CE (T2) → PCPR (T3): standardized indirect effect = 0.065, SE = 0.018, 95% CI = (0.032, 0.10)], supporting H4. These findings provide longitudinal predictive evidence for the proposed theory-guided behavior–identity–efficacy pathway linking community-based sport-leisure participation to perceived community psychological resilience.

## 5 Discussion

### 5.1 Discussion of main findings

Using three-wave longitudinal data and an RI-CLPM, this study examined prospective within-person associations among community-based sport-leisure participation, community identification, collective efficacy, and perceived community psychological resilience. The overall pattern was consistent with the proposed behavior–identity–efficacy pathway: participation above a resident’s own typical level predicted later resilience-related perceptions, and the model-defined indirect associations through community identification, collective efficacy, and their sequential combination were statistically significant. These results are interpreted as longitudinal predictive associations rather than causal effects.

The contribution of the findings lies primarily in specifying a temporally ordered social-psychological pattern while separating stable between-person differences from within-person fluctuations. This reduces the possibility that the associations merely reflect generally more sociable residents reporting higher scores on all constructs. At the same time, the magnitude of the effects warrants a calibrated interpretation. The focal direct paths were modest (β = 0.18 from T1 to T2 and *β* = 0.21 from T2 to T3), and the model-defined indirect effects ranged from 0.065 to 0.098. Community-based sport-leisure participation should therefore be viewed as one potentially relevant predictor among multiple social, organizational, and contextual influences on perceived community resilience.

For H1, higher-than-usual sport-leisure participation prospectively predicted higher perceived community psychological resilience at the next wave. This pattern is compatible with prior research linking sport and leisure participation with positive affect, subjective well-being, and social benefits (Eime et al., 2013; Sato et al., 2018; Wicker and Frick, 2015), but the present study extends that evidence only in a limited sense: it shows that within-person variation in a community-based behavioral practice is associated with later variation in residents’ appraisals of their community. It does not establish that increasing participation will necessarily produce greater resilience.

The indirect results further indicate that community identification and collective efficacy capture distinguishable parts of this prospective pattern. Community identification concerns whether residents experience the community as part of a meaningful ‘we,’ whereas collective efficacy concerns whether that ‘we’ is perceived as capable of coordinated action. The sequential indirect effect is therefore theoretically informative because it links affective belonging with agency-related beliefs, while its modest size cautions against treating this pathway as exhaustive.

Taken together, the findings are most appropriately read as evidence consistent with a theory-guided longitudinal process rather than as confirmation of a deterministic mechanism. Unmeasured time-varying factors, differences in activity quality, and broader neighborhood conditions may also influence the observed associations.

### 5.2 Sport-leisure participation as informal social infrastructure and a context for community identification

The social-infrastructure perspective clarifies what is distinctive about community-based sport-leisure participation. Unlike physical activity performed in isolation, neighborhood walking groups, public fitness activities, square dancing, running groups, and informal ball games can create recurring opportunities for co-presence in familiar local spaces. These opportunities may increase residents’ exposure to neighbors, shared routines, and informal interaction, making sport-leisure participation a plausible social context in which community-related perceptions are formed (Latham and Layton, 2022; Nicholson and Hoye, 2008; Zhou and Kaplanidou, 2018).

Social Identity Theory adds a more specific psychological interpretation. Repeated co-presence does not automatically create belonging, but it can make community membership more salient and provide occasions for mutual recognition. The positive prospective association between sport-leisure participation and community identification is consistent with the possibility that participation is one setting in which residential proximity becomes psychologically meaningful. This interpretation aligns with work on sense of community and place attachment, which emphasizes shared experience, emotional connection, and social meaning (McMillan and Chavis, 1986; Peterson et al., 2008; Scannell and Gifford, 2010; Lewicka, 2011).

Importantly, this interpretation has boundary conditions. Participation that is involuntary, highly competitive, exclusionary, poorly organized, or inaccessible to some residents may provide little basis for shared belonging and could even reinforce social distance. Likewise, low-quality or unsafe sport-leisure spaces may limit repeated interaction. Thus, the relevant theoretical point is not that sport participation itself produces identification, but that inclusive and socially meaningful participation may provide opportunities for identification to develop.

This distinction also reduces the risk of overstating the notion of sport-leisure participation as ‘social infrastructure.’ The present data capture residents’ reported participation and perceptions; they do not demonstrate that sport facilities or programs function as social infrastructure in every community. Future studies should directly assess activity quality, inclusiveness, interaction density, and accessibility to determine when this interpretation is most applicable.

### 5.3 Collective efficacy and the scope of the behavior–identity–efficacy pathway

Collective efficacy contributes a conceptually distinct step to the proposed pathway. Community identification addresses the question ‘Do I belong to this community?’, whereas collective efficacy addresses ‘Can we act together as a community?’ Residents may feel attached to a neighborhood without being confident that neighbors can coordinate responses to shared problems. The positive lagged association from identification to collective efficacy is therefore consistent with the idea that a stronger sense of ‘we’ may be accompanied by stronger beliefs about collective capability.

Social Cognitive Theory provides the relevant agency-based account. Everyday sport-leisure settings can involve rule-following, sharing space, minor coordination, and observation of others’ cooperation. Such experiences may serve as cues from which residents infer whether collective action is feasible, although the present design cannot establish that these experiences caused the observed changes in efficacy beliefs (Bandura, 1990, 1997, 2000; Sampson et al., 1997).

The association between collective efficacy and later perceived community psychological resilience also helps define the scope of the outcome. The study concerns residents’ appraisals of whether their community is connected, capable, and psychologically prepared; it does not measure objective emergency capacity, infrastructure, or institutional performance. The results therefore suggest that everyday social participation may be one context associated with resilience-related perceptions before crises occur, rather than demonstrating that sport-leisure participation creates objective community resilience.

This narrower interpretation still connects behavioral science, community psychology, and resilience research. The behavior–identity–efficacy sequence offers a process-oriented account of how an individual behavioral context can be prospectively associated with community-level psychological appraisals, but the small-to-moderate effects indicate that the pathway is partial. Structural resources, governance quality, social inequality, prior trust, and other forms of community participation are likely to operate alongside it.

The pathway may also be weaker or absent in communities facing socioeconomic disadvantage, limited public space, low baseline trust, persistent conflict, or unequal access to activities. Under such conditions, opportunities for repeated participation may not translate into belonging or shared efficacy. Testing these boundary conditions is important for determining the generalizability of the proposed sequence.

### 5.4 Methodological implications of the RI-CLPM approach

The RI-CLPM strengthens the longitudinal interpretation by separating stable between-person differences from within-person fluctuations (Hamaker et al., 2015; Orth et al., 2021). This is important here because residents who are generally more socially active may report higher participation, identification, efficacy, and resilience at every wave. The within-person estimates instead address whether deviations from a resident’s own typical level at one wave prospectively predict deviations in another construct at the next wave.

The method does not, however, convert observational data into causal evidence. Unmeasured time-varying confounders and reciprocal processes may remain even after random intercepts, autoregressive paths, and alternative-model comparisons are considered. The statistically significant coefficients should also be interpreted in light of their modest magnitude: the focal direct effect was *β* = 0.18 at T1 → T2 (*β* = 0.21 at T2 → T3), while the indirect effects were 0.098, 0.085, and 0.065. These estimates support the presence of prospective associations but not a large or dominant behavioral effect.

Future studies could strengthen inference through four or more waves, preregistered temporal models, objective participation records, ecological momentary assessment, and intervention or quasi-experimental designs. Such approaches would allow researchers to test whether experimentally or naturally occurring changes in participation are followed by changes in identification, efficacy, and resilience-related perceptions and whether the timing of those changes matches the proposed sequence.

### 5.5 Practical implications

The practical implications should be considered as hypotheses for community practice rather than as causal prescriptions. The present findings do not show that expanding sport-leisure programs will by itself increase community resilience. Instead, they suggest that community-based sport-leisure activities may be useful settings for social contact, belonging, and perceived collective capability when programs are accessible, inclusive, and embedded in everyday neighborhood life.

This interpretation is particularly relevant to urban China, where community governance and national fitness promotion increasingly intersect at the neighborhood level. Local governments, neighborhood committees, community managers, and sport organizers can consider not only the provision of exercise opportunities but also whether public sport-leisure spaces support repeated, low-threshold interaction among residents. In communities that already have basic facilities, programming that encourages shared participation across age groups and ability levels may align national fitness goals with broader community-building objectives.

Program design should therefore prioritize accessibility and social quality rather than athletic performance alone. Walking groups, public fitness activities, family-oriented events, and interest-based resident groups may provide opportunities for coordination and familiar interaction. Because the empirical effects were modest, such programs are best viewed as one component of a broader community-governance strategy that also includes social services, volunteer networks, public-space quality, and institutional preparedness.

The distinction between resource-endowed and under-resourced communities is especially important. Where safe spaces, equipment, or organizational support are limited, recommending greater participation without first addressing infrastructure and access may be unrealistic. Practical application should therefore be adapted to local resource conditions rather than generalized uniformly across communities.

### 5.6 Limitations and future research

Several limitations should be acknowledged. First, all core variables were measured using residents’ self-reports. Although the RI-CLPM separated stable response tendencies and the common-method checks did not indicate a substantial method-factor problem, future studies should combine self-reports with objective behavioral records, observational indicators, administrative data, or multi-source assessments. Second, the three-wave design strengthens temporal ordering but does not establish causality; the indirect effects should be interpreted as theory-guided longitudinal associations rather than as proof of psychological mechanisms.

Third, attrition was substantial. Of the 800 participants at baseline, 386 provided fully matched data across all three waves, corresponding to a retention rate of 48.25% and an attrition rate of 51.75%. Attrition is a recurring challenge in community-based longitudinal research, but the magnitude observed here is not trivial. Baseline comparisons and Little’s MCAR test did not identify systematic differences between retained and lost participants on the measured variables, yet these tests cannot exclude selection on unmeasured characteristics. The reduced fully matched sample may therefore limit statistical precision and generalizability.

Fourth, the three study communities were selected because they already had established sport-leisure facilities and regular recreational activities. This sampling feature creates a meaningful boundary on external validity: the observed associations may be more applicable to urban communities with an existing level of sport-related infrastructure and resident participation than to under-resourced neighborhoods. Replication should include communities with different levels of facility provision, organizational capacity, socioeconomic resources, and baseline participation.

Fifth, all four instruments originated in Western research contexts. The present study used translation/back-translation and conservative context-specific adaptation, and longitudinal measurement invariance supported temporal comparability within this sample. Nevertheless, future Chinese studies should add formal expert content-validity assessment, cognitive interviewing or pilot testing, and where possible cross-cultural invariance tests to provide stronger evidence for cultural equivalence.

Finally, the data were collected in urban communities in eastern China and the outcome was perceived rather than objective community resilience. Future work should test cross-cultural generalizability, combine residents’ perceptions with indicators such as emergency resources, organizational networks, volunteer participation, and public-space availability, and explicitly examine boundary conditions including activity quality, voluntariness, social inclusion, neighborhood socioeconomic status, baseline trust, age, gender, and social support.

## 6 Conclusion

Based on an 8-month, three-wave longitudinal dataset, this study found that within-person increases in community-based sport-leisure participation prospectively predicted later perceived community psychological resilience, with significant model-defined indirect associations through community identification and collective efficacy. The pattern is consistent with a behavior–identity–efficacy pathway linking an everyday behavioral context with social belonging, perceived collective capability, and resilience-related appraisals. Because the effects were modest and the design was observational, community sport-leisure participation should be regarded as one potentially relevant social-psychological context rather than as a demonstrated causal lever for resilience. This calibrated interpretation highlights a useful connection between sport-leisure behavior and community psychology while identifying clear conditions for future replication and stronger causal testing.

## Data Availability

The raw data supporting the conclusions of this article will be made available by the authors, without undue reservation.
